# Combined *Taraxacum mongolicum* and Astragalus polysaccharides attenuate CCl_4_-induced hepatic fibrosis through modulation of the TLR4/MyD88/NF-κB signaling pathway and gut microbiota composition

**DOI:** 10.3389/fphar.2026.1904671

**Published:** 2026-09-17

**Authors:** Yingjie Ma, Chaoxing Wang, Xue Chen, Shiqing Sun, Shuang Li, Baisong Guo, Wenda Wang, Yu Shen, Yu Zhang, Yuliang Wang

**Affiliations:** 1 College of Pharmacy, Jiamusi University, Jiamusi, China; 2 College of Basic Medicine, Jiamusi University, Jiamusi, China

**Keywords:** Astragalus polysaccharide, gut microbiota, gut-liver axis, hepatic fibrosis, *Taraxacum mongolicum* polysaccharide, TLR4/MYD88/NF-κB signaling pathway

## Abstract

**Background:**

Hepatic fibrosis represents a critical pathological stage in the progression of chronic liver diseases, yet clinically effective and low-toxicity therapeutic options remain limited. *Taraxacum mongolicum* and Astragalus form a classic hepatoprotective herbal pair, and their derived polysaccharides-*Taraxacum mongolicum* polysaccharides (TMP) and Astragalus polysaccharides (APS)-both exhibit hepatoprotective activities. This study aimed to investigate the ameliorative effects of TMP combined with APS on CCl_4_-induced hepatic fibrosis in mice and the gut-liver axis-associated changes.

**Methods:**

Mice were randomly assigned to the blank control, model, silybin, Lizhu Changle (live Bifidobacterium adolescentis capsules), TMP monotherapy, APS monotherapy, and combination groups. Following the establishment of fibrosis by CCl_4_, a 4-week intervention was administered. Serum liver function parameters and fibrosis markers were measured, and hepatic histopathological changes were observed. Intestinal function was comprehensively evaluated. Gut microbiota composition was analyzed using 16S rRNA gene sequencing, and fecal short-chain fatty acid (SCFA) levels were quantified by high-performance liquid chromatography (HPLC). The protein expression of the hepatic TLR4/MyD88/NF-κB pathway was further assessed.

**Results:**

Compared with TMP or APS alone, the combination significantly improved multiple fibrosis-related, inflammatory, intestinal barrier, microbial diversity, and SCFA-related indices, although significant superiority was not observed for PC III and TNF-α. The combination also more strongly suppressed hepatic TLR4/MyD88/NF-κB pathway expression.

**Conclusion:**

TMP combined with APS exerts enhanced anti-hepatic fibrosis effects compared with either monotherapy. These effects are associated with changes in gut microbiota composition, elevated fecal short-chain fatty acid levels, reduced expression of hepatic TLR4/MyD88/NF-κB pathway-related molecules. These findings provide an experimental basis for further investigation of this herbal pair.

## 1 Introduction

Hepatic fibrosis (HF), a pathological process marked by excessive extracellular matrix deposition during the wound-healing response to chronic liver injury, serves as a pivotal driver of progression from various chronic liver diseases to cirrhosis and hepatocellular carcinoma (Sun et al., 2025; Nishikawa et al., 2026). Globally, the incidence and mortality of HF-associated conditions continue to rise, imposing a substantial public health burden (Kuang et al., 2026; Li et al., 2026). Current clinical management relies heavily on etiological control, such as antiviral therapy and alcohol abstinence. Although these approaches can slow disease progression, they exhibit significant shortcomings in reversing established fibrosis, ensuring long-term medication safety, and addressing liver diseases that lack specific etiological treatments (Xie and Singal, 2023; Liu et al., 2024; Zhang et al., 2026). Consequently, the pursuit of novel antifibrotic strategies that offer multi-target intervention, high potency, low toxicity, and suitability for chronic administration has become an urgent imperative in hepatology research.

In recent years, traditional Chinese medicine (TCM) and its bioactive constituents have demonstrated substantial potential in preventing and treating hepatic fibrosis (HF), largely owing to their multi-target and holistic regulatory properties (Li, 2020; Yang et al., 2021; Bai et al., 2024). Among them, polysaccharides, an important category of natural macromolecules, have garnered extensive interest owing to their potent anti-inflammatory, immunomodulatory and organ-protective effects (Tang et al., 2018; Yuan et al., 2019). Notably, accumulating evidence shows that Cordyceps polysaccharide and fucoidan can ameliorate HF through antioxidant defense, inhibition of hepatic stellate cell activation, and modulation of the gut microbiota (Liu et al., 2013; Li et al., 2016; Li et al., 2022; Zhao et al., 2022). These findings highlight a broad prospect for screening and developing anti-HF agents from natural polysaccharides.

*Taraxacum mongolicum* and Astragalus form a classic herbal pair in traditional Chinese medicine for treating liver disorders. The application of this herbal pair embodies the therapeutic principle of “clearing heat and detoxifying, replenishing qi and reinforcing the body’s resistance,” and clinicians have widely adopted this pairing in practice. Modern studies have confirmed that *Taraxacum mongolicum* polysaccharide exhibits hepatoprotective activities, including antioxidant and anti-inflammatory effects (Cai et al., 2019; Zhou S. et al., 2023). Research has also shown that Astragalus polysaccharide can inhibit hepatic stellate cell proliferation and induce apoptosis, and that combining it with β-elemene enhances this efficacy (Zheng et al., 2014; Hamid et al., 2017). However, existing investigations have largely concentrated on the effects of individual herbs. Whether this herbal pair exerts complementary anti-fibrotic effects and outperforms monotherapy, as well as the molecular mechanisms by which it regulates the gut-liver axis, remain to be systematically verified. Notably, leveraging the compatibility of different bioactive components or polysaccharides to achieve synergistic potentiation has emerged as a key strategy in natural product development. Studies have demonstrated that Astragalus polysaccharide and astragalosides, active constituents of Astragalus, can protect against alcoholic liver injury by modulating the gut microbiota and attenuating oxidative stress and inflammation (Zhou et al., 2021). More directly, evidence has confirmed that the combination of sea buckthorn polysaccharide and Astragalus polysaccharide synergistically regulates the gut microbiota and improves liver function parameters in mice with alcoholic fatty liver disease (Liu et al., 2022). Therefore, for hepatic fibrosis—a complex pathological process driven by multiple factors—the rational combination of *Taraxacum mongolicum* polysaccharide and Astragalus polysaccharide holds considerable promise for achieving superior therapeutic efficacy through multi-target intervention.

Among the multiple pathological mechanisms driving hepatic fibrosis, gut-liver axis dysfunction has gained increasing prominence and now stands as a critical nexus linking intestinal homeostasis to hepatic inflammation and fibrogenesis (Zhou C. et al., 2023; Li X. et al., 2024; Ding et al., 2026). Gut dysbiosis and intestinal barrier disruption permit endotoxins, such as lipopolysaccharide (LPS), to translocate into the liver (Su et al., 2023), where they engage hepatic Toll-like receptor 4 (TLR4) and activate the downstream myeloid differentiation factor 88 (MyD88)/nuclear factor-κB (NF-κB) classical inflammatory cascade. This process persistently fuels hepatic stellate cell activation and drives fibrotic progression (Yu et al., 2006; Liu et al., 2020; Zheng et al., 2024; Yuan et al., 2025). These insights identify the gut-liver axis as an attractive therapeutic target for combating hepatic fibrosis. Notably, studies have demonstrated that colon-targeted delivery of Astragalus polysaccharide exerts protective effects in models of nonalcoholic fatty liver disease (NAFLD) and NAFLD-related hepatocellular carcinoma. This protection arises from reshaping the gut microbiota, boosting the production of short-chain fatty acids such as acetate, and consequently inhibiting the hepatic NF-κB signaling pathway (Liu et al., 2025). In parallel, emerging evidence indicates that *Taraxacum mongolicum* polysaccharide also holds considerable promise in modulating gut microbiota composition and intestinal barrier integrity (Cao et al., 2023; Yan and Dong, 2024).

Therefore, using a CCl_4_-induced mouse model of hepatic fibrosis, this study compares the interventional effects of this herbal pair (TMP and APS) administered alone and in combination. We specifically aim to evaluate the anti-fibrotic effects of this polysaccharide combination and, from the perspective of the gut-liver axis, to characterize the associated changes in microbial metabolism, endotoxin-related markers, and the TLR4/MyD88/NF-κB signaling pathway.

## 2 Materials and methods

### 2.1 Materials and reagents

Dextran standards (12,000, 25,000, 50,000, 80,000, 150,000, and 270,000 Da) were purchased from Sigma (St. Louis, MO, United States). Ten monosaccharide standards, including D-mannose (Man), D-ribose (Rib), L-rhamnose (Rha), D-glucuronic acid (GlcA), D-galacturonic acid (GalA), D-glucose (Glc), D-galactose (Gal), D-xylose (Xyl), D-arabinose (Ara), and D-fucose (Fuc), were also obtained from Sigma (St. Louis, MO, United States). Silymarin capsules (Shuilinjia, 35 mg per capsule) were acquired from Tasly Pharmaceutical Group Co., Ltd. (Tianjin, China). Acetic acid-aniline blue staining solution, hematoxylin-eosin (H&E) staining solution, and Ponceau-fuchsin staining solution were all sourced from Wuhan Servicebio Biotechnology Co., Ltd. (Wuhan, China). Anti-MyD88 antibody (batch no. 20210616), anti-NF-κB p65 antibody (batch no. 20210616), and anti-TLR4 antibody (batch no. 20210616) were procured from Beijing Tiande Rui Biotechnology Co., Ltd. (Beijing, China). The DNA detection kit was obtained from Invitrogen (Carlsbad, CA, United States).

### 2.2 Extraction and purification of *Taraxacum mongolicum* polysaccharide and Astragalus polysaccharide

The dried whole plants of *Taraxacum mongolicum* Hand.-Mazz. were purchased from Tongrentang Pharmacy (Jiamusi, Heilongjiang, China; batch No. 170801) and authenticated as *Taraxacum mongolicum* Hand.-Mazz. (Asteraceae). Dried roots of *Astragalus membranaceus* (Fisch.) Bge. var. *mongholicus* (Bge.) Hsiao were purchased from Zhanqian Pharmacy (formerly Minsheng Pharmacy, Jiamusi, Heilongjiang, China; batch No. 181006) and authenticated as Astragalus membranaceus (Fisch.) Bge. var. mongholicus (Bge.) Hsiao (Fabaceae). The dried *T. mongolicum* plants were boiled in water at a ratio of 1:30 (w/v) for 2 h and filtered while hot. The supernatant was collected and concentrated under reduced pressure, and polysaccharides were precipitated by the addition of ethanol for 24 h. The precipitate was collected, re-dissolved, and freeze-dried, and the resulting product was stored at −80 °C. For *A. membranaceus*, the dried root slices were crushed into small pieces and defatted by ethanol reflux. The defatted residue was boiled in water at a ratio of 1:8 (w/v) and filtered, and the filtrate was concentrated under reduced pressure. After ethanol precipitation for 24 h, the precipitate was freeze-dried to obtain crude Astragalus polysaccharide. The crude *Taraxacum mongolicum* polysaccharide and crude Astragalus polysaccharide were each deproteinized with trichloroacetic acid, dialyzed against running water for 48 h, and subsequently freeze-dried to yield deproteinized *Taraxacum mongolicum* polysaccharide (TMP) and Astragalus polysaccharide (APS), respectively.

S-8 macroporous adsorption resin was selected and packed into a column by the wet method, after which the column was equilibrated with four bed volumes of distilled water. The deproteinized *Taraxacum mongolicum* polysaccharide was dissolved in distilled water to a concentration of 5 mg mL^−1^, and the resulting solution was centrifuged. The supernatant was transferred onto the column and allowed to adsorb for 8 h, after which the eluate was collected and concentrated under reduced pressure. The concentrate was dialyzed against running water for 48 h and subsequently freeze-dried to yield purified *Taraxacum mongolicum* polysaccharide, designated as TMP. Employing the same procedures with HP-20 macroporous resin, the deproteinized Astragalus polysaccharide was purified and vacuum freeze-dried to obtain purified Astragalus polysaccharide, designated as APS.

### 2.3 Physicochemical characterization of purified TMP and APS

The polysaccharide content was determined by the phenol-sulfuric acid method using glucose as the standard, and the protein content was measured by the Bradford method with bovine serum albumin as the standard. Both purified polysaccharides, TMP and APS, were scanned using a 765 UV-Vis spectrophotometer. The purified TMP and APS samples were pressed into KBr pellets and scanned with an infrared spectrometer over the range of 4,000–5,000 cm^−1^. The relative molecular weights of the purified polysaccharides were determined by high-performance gel permeation chromatography (HPGPC). The liquid chromatography system employed was an LC-5510 high-performance liquid chromatography (HPLC) system (Beijing East & West Analytical Instruments Co., Ltd., Beijing, China) equipped with a UM4800 evaporative light scattering detector (ELSD). Monosaccharide composition analysis was performed using an Agilent 1260 HPLC system (Agilent Technologies, Santa Clara, CA, United States). Detailed information regarding instrument parameters and sample preparation is provided in the Supplementary Data. The surface morphology and characteristics of the polysaccharide samples were observed with a Phenom ProX scanning electron microscope (SEM; Phenom-World, Eindhoven, Netherlands). The crystalline structure of the polysaccharide samples was assessed using an X-ray diffractometer (XRD).

### 2.4 Animal models, experimental design and ethical approval

SPF-grade male Kunming mice (8 weeks old) were obtained from Changchun Yisi Laboratory Animal Technology Co., Ltd. (Changchun, China; license No. SCXK (Ji) 2020-0002). The animals were housed under controlled environmental conditions at a temperature of 22 °C ± 2 °C, a relative humidity of 40%–60%, and a 12-h light/dark cycle. All animal experimental procedures were conducted in strict accordance with the ethical guidelines of the Chinese Association for Laboratory Animal Sciences, and humane care was provided throughout the experiments. This study was approved by the Animal Ethics Committee (Approval No. JMSU-234).

After 1 week of acclimatization, hepatic fibrosis was induced in mice by intraperitoneal injection of carbon tetrachloride (CCl_4_). Specifically, CCl_4_ was mixed with olive oil at a volume ratio of 1:3 to prepare the modeling agent. Except for mice in the blank control group (Con), which received an equivalent volume of pure olive oil, mice in all other groups were intraperitoneally injected with the CCl_4_-olive oil solution at 2 mL/kg body weight, three times per week for eight consecutive weeks.

Mice were randomly divided into seven groups (n = 10 per group): the blank control group (Con) and the model group (Mod) were intragastrically administered an equivalent volume of normal saline (10 mL/kg); the positive control groups received silymarin (Pos1, 100 mg/kg) or live Bifidobacterium preparation (Pos2, 90 mg/kg, a commercial probiotic preparation containing live Bifidobacterium adolescentis), respectively; and the experimental groups received *Taraxacum mongolicum* polysaccharide (TMP, 200 mg/kg), Astragalus polysaccharide (APS, 200 mg/kg), or their combination (TMP + APS, total dose 200 mg/kg, consisting of 100 mg/kg TMP and 100 mg/kg APS). All treatments were initiated 4 weeks after the start of CCl_4_ injection and were administered once daily by gavage for 4 weeks.

### 2.5 Sample collection and histological scores

Ileal tissues were used to evaluate mucosal architecture and inflammatory alterations, while colonic contents/feces were employed to reflect the microbial community composition and metabolite levels. At the end of the experiment, fecal and serum samples were immediately collected and stored at −80 °C for subsequent analyses. Body weight was recorded weekly, and the changes were analyzed. The liver weight of non-perfused mice and the body weight at week 9 were measured, and the liver index was calculated using the formula: liver index = liver wet weight (mg)/body weight (g). After sacrifice, liver and ileal tissues from each mouse were divided into two aliquots for different analyses. One aliquot was collected from transcardially perfused mice, fixed in 4% paraformaldehyde, embedded in paraffin and sectioned at 5 μm for histological staining. The other aliquot was harvested from non-perfused mice and stored at −80 °C for ELISA, Western blot and qRT-PCR analyses. Tissues for histological observation were obtained from transcardially perfused mice to remove residual blood and avoid interference with morphological examination. For the semi-quantitative analysis of collagen in Masson-stained sections, three mice were used per group. Five non-overlapping fields were randomly selected from each section for image acquisition. The proportion of collagen area to the total tissue area was calculated using ImageJ software via color threshold segmentation. All samples were de-identified with respect to their group labels, and the quantitative image analysis was conducted in a single-blind manner. This study described tissue damage solely based on microscopic morphological observations and did not employ a pathological grading or scoring system. Samples for molecular and biochemical detection were collected without perfusion to preserve endogenous soluble factors and proteins. Concurrently, colonic contents were collected from each mouse, placed in sterile Eppendorf tubes, and stored at −80 °C pending 16S rRNA gene sequencing. For 16S rRNA gene sequencing, samples were collected from the colon contents of 10 mice in each group, and samples that did not meet the DNA quality standards were excluded. Short-chain fatty acid (SCFA) samples (n = 6 per group) were obtained from the mice whose colon contents were used for microbiota analysis, allowing for paired correlation analysis. The fecal samples used for SCFA detection (6 samples per group) were randomly selected from the same batch of mice that underwent 16S sequencing, thus ensuring the pairing of the correlation analysis at the individual level.

### 2.6 Enzyme-linked immunosorbent assay

The levels of alanine aminotransferase (ALT), aspartate aminotransferase (AST), hyaluronic acid (HA), procollagen type III (PC III), and collagen type IV (Col IV) in serum, the concentrations of lipopolysaccharide (LPS), interleukin-6 (IL-6), and tumor necrosis factor-alpha (TNF-α) in liver tissue, and the content of secretory immunoglobulin A (sIgA) in ileal tissue were measured using enzyme-linked immunosorbent assay (ELISA) kits (Nanjing Jiancheng Bioengineering Institute, Nanjing, China) strictly according to the manufacturer’s instructions.

### 2.7 Analysis of gut microbiota diversity

DNA was extracted from the colonic contents of mice, and the extracts were subjected to quantification and quality assessment using a Nanodrop spectrophotometer (Thermo Fisher Scientific, Wilmington, DE, United States) and 1.2% agarose gel electrophoresis. After confirming that the bands met the quality requirements, the target fragments were PCR-amplified for subsequent data analysis. The V3-V4 hypervariable region of the bacterial 16S rRNA gene was amplified with the primer pair 338F (5′-ACT​CCT​ACG​GAG​GCA​GCA-3′) and 806R (5′-GGA​CAC​TCG​GTC​CGT​CGT-3′), and the recovered amplification products were quantified by fluorometry and qualified using the TruSeq Nano DNA LT Library Prep Kit (Illumina, San Diego, CA, United States). High-throughput sequencing of the colonic content samples was performed on the NovaSeq 6000 platform (Illumina). The relative abundance data derived from 16S rRNA sequencing were compared among groups by the Kruskal–Wallis test, with a *P* value < 0.05 considered statistically significant. The positive control groups (Pos1 and Pos2) were included in the pharmacological efficacy analyses to validate model responsiveness but were excluded from the gut microbiota mechanistic analysis to avoid potential confounding, as silymarin and Bifidobacterium may act through mechanisms distinct from TMP/APS. Linear discriminant analysis effect size (LEfSe) analysis was further performed to identify differential microbial biomarkers across experimental groups, and pairwise LEfSe comparison was separately conducted between the blank control group and the model group. The Kruskal–Wallis rank-sum test and Wilcoxon signed-rank test (alpha = 0.05) were used to screen taxa with significant intergroup differences, and the linear discriminant analysis (LDA) score threshold was set to 2.0.

### 2.8 Determination of short-chain fatty acids in mouse feces

Six fecal samples from each group were randomly selected for pretreatment. The supernatant was collected after centrifugation and filtered through a 0.22-μm aqueous-phase filter, and subsequent detection was carried out using an Agilent 1260 HPLC system (Agilent Technologies, Santa Clara, CA, United States) equipped with a Carbomix H-NP5:8% column (7.8 mm × 300 mm, 5 μm). Standard curves were constructed by plotting the concentrations (g·100 mL^−1^) of short-chain fatty acids (SCFAs) against their corresponding peak areas. All seven SCFAs (formic acid, acetic acid, propionic acid, n-butyric acid, isobutyric acid, n-valeric acid, and isovaleric acid) exhibited excellent linearity within their respective concentration ranges, with correlation coefficients (*R* ^2^) ≥ 0.9990. Detailed instrument parameters and procedures are provided in the Supplementary Data.

### 2.9 Western blot and qRT-PCR

Liver tissues obtained from non-perfused mice were homogenized in lysis buffer containing phenylmethylsulfonyl fluoride (PMSF) and subjected to ultrasonication to extract total protein lysates. The proteins were separated by sodium dodecyl sulfate-polyacrylamide gel electrophoresis (SDS-PAGE) and transferred onto polyvinylidene difluoride (PVDF) membranes using the wet transfer method. The membranes were blocked with 5% non-fat dry milk for 90 min and subsequently incubated with primary antibodies against GAPDH (1:20,000), TLR4 (1:2,000), MyD88 (1:1,000), and NF-κB p65 (1:1,000) on a shaker at 4 °C overnight. On the following day, the membranes were incubated with the corresponding secondary antibody (1:5,000) at room temperature for 90 min with shaking. Enhanced chemiluminescence (ECL) detection was performed, and the blots were exposed. Grayscale values were quantified using ImageJ software (version 1.8.0, National Institutes of Health, United States).

Total RNA was extracted from non-perfused liver tissues. The RNA concentration and purity were determined using a Nanodrop 2000 spectrophotometer (Thermo Fisher Scientific, Wilmington, DE, United States), and the RNA was reverse transcribed into cDNA. Quantitative real-time PCR (qRT-PCR) analysis was performed on a StepOne Plus Real-Time PCR System (Applied Biosystems, Foster City, CA, USA) using SYBR Green Master Mix (Applied Biosystems). Relative gene expression levels were calculated using the 2^−ΔΔCT^ method. The primer sequences employed are listed in Table 1.

**TABLE 1:** Primers for quantitative real-time PCR.

Primer information	Primer sequence	Product size (bp)
NM_021297.2 TLR4	5′-TGAGGACTGGGTGAGAAATGAGC-3′ 5′-CTGCCATGTTTGAGCAATCTCAT-3′	223
NM_021297.2 MyD88	5′-CGATGCCTTTATCTGCTACTGC-3′ 5′-GCTTCTGTTGGACACCTGGAG-3′	269
NM_001365067.1 NF-*κ*B p65	5′-CGAGTCTCCATGCAGCTACG-3′ 5′-TTTCGGGTAGGCACAGCAATA-3′	212
NM_008084.2 GAPDH	5′-CCTCGTCCCGTAGACAAAATG-3′ 5′-TGAGGTCAATGAAGGGGTCGT-3′	133

### 2.10 Statistical analyses

All data were expressed as the mean ± standard deviation (SD) of at least three independent experiments. All *in vivo* indices (serum biochemical, histological, inflammatory, gut microbiota, SCFA, and pathway protein/mRNA indicators) were derived from biological replicates (individual mice per group). For *in vitro* polysaccharide characterization tests, data came from three independent repeated measurements. Normality was assessed by the Shapiro-Wilk test, and homogeneity of variances was verified by Levene’s test. One-way analysis of variance (ANOVA) followed by Tukey’s HSD test was applied for data meeting parametric assumptions. For data not meeting these assumptions, the Kruskal–Wallis test with Dunn’s *post hoc* test was used. The *p* value < 0.05 was considered statistically significant. In addition to comparisons with the model group, prespecified pairwise comparisons were performed between the TMP + APS group and each monotherapy group (TMP and APS). The corresponding significance levels are indicated in the figures. Spearman’s correlation analysis was conducted to evaluate the correlations among hepatic signaling pathway activity, gut microbiota composition, inflammatory cytokine levels, and short-chain fatty acid concentrations. Statistical analyses were performed using SPSS Statistics 27 (IBM, United States).

## 3 Results

### 3.1 Physicochemical characterization of TMP and APS

Chemical composition analysis demonstrated that the total sugar contents of purified TMP and APS were 79.72% ± 0.45% and 79.01% ± 0.81%, respectively, while their protein contents were 0.53% ± 0.08% and 0.49% ± 0.05%, respectively. All TMP and APS used in this study were obtained from one batch of large-scale extraction and purification. All cellular and animal intervention experiments were performed using this identical batch of polysaccharides to eliminate experimental interference caused by batch-to-batch variation. The extraction yields of TMP and APS are detailed in the supplementary materials.

The UV spectra of the purified polysaccharides TMP and APS are presented in Figure 1A. No prominent absorption peaks at 260–280 nm were found for purified TMP and APS, implying low residual nucleic acid and protein impurities.

**FIGURE 1:**
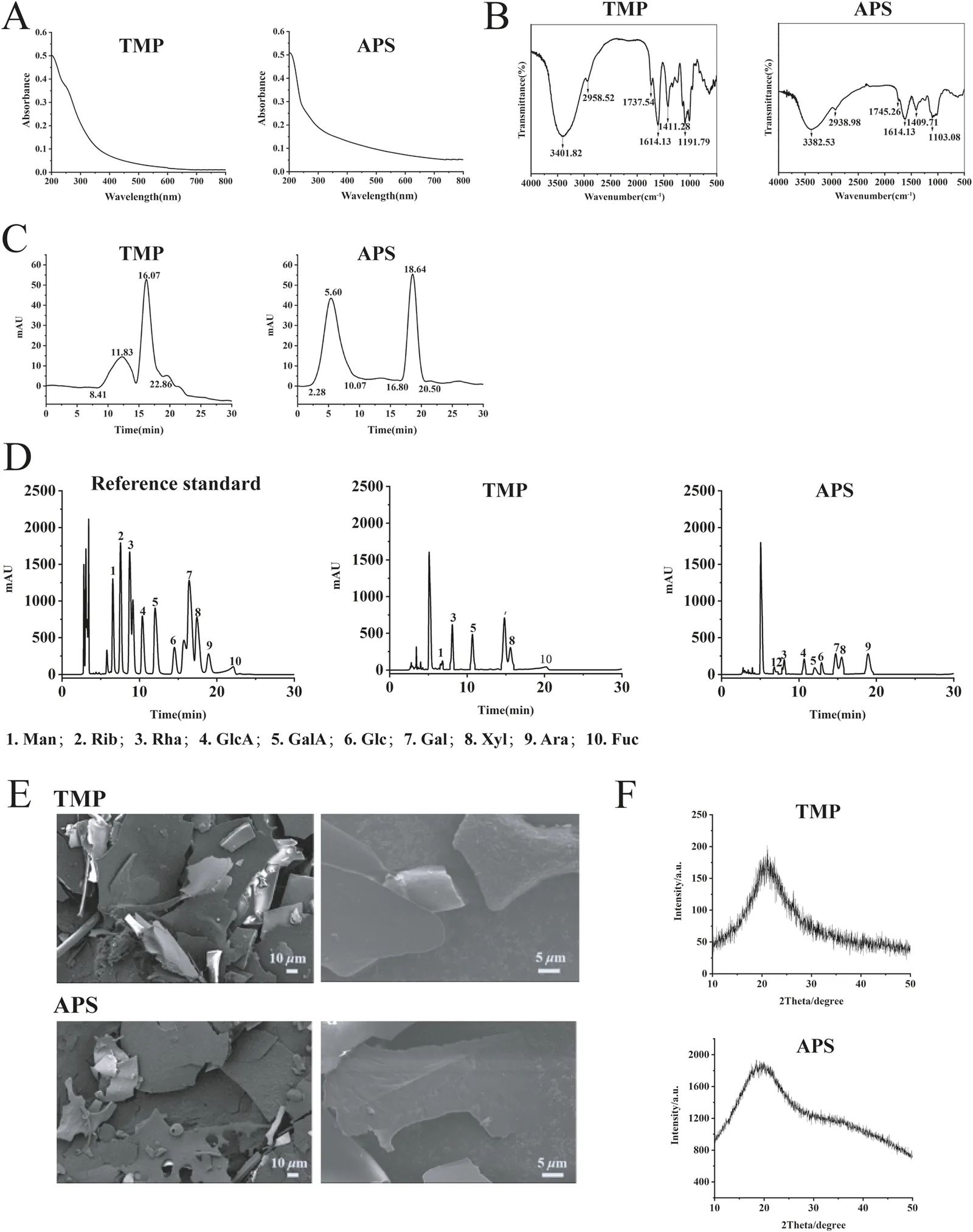
Physicochemical characterization of *Taraxacum mongolicum* Polysaccharides (TMP) and Astragalus Polysaccharides (APS). **(A)** UV-vis spectra. **(B)** FT-IR spectra. **(C)** Molecular weight distribution determined by HPGPC. **(D)** Monosaccharide composition analysis. **(E)** SEM images. **(F)** XRD patterns.

The FT-IR spectra of TMP and APS were recorded in the wavenumber range of 4,000–500 cm^−1^ (Figure 1B), which could only identify characteristic functional groups of polysaccharides, rather than elucidate their complete molecular structures. A broad absorption band near 3,400 cm^−1^ in both samples was assigned to the stretching vibration of O-H bonds. The characteristic peaks at 2958.52 and 2938.98 cm^−1^ originated from C-H stretching vibrations, which are universal signature signals of polysaccharide macromolecules. Moreover, the absorption peaks at 1737.54 cm^−1^ and 1745.26 cm^−1^ may tentatively suggest the presence of asymmetric C=O stretching vibrations, which are speculated to originate from ester carbonyl groups within the polysaccharide skeleton.

High-performance gel permeation chromatography (HPGPC) analysis revealed that both purified *Taraxacum mongolicum* polysaccharide and purified Astragalus polysaccharide exhibited two major chromatographic peaks (Figure 1C). The molecular weight distribution of TMP consisted of two fractions with molecular weights of 4.85 × 10^5^ Da and 7.08 × 10^4^ Da, respectively. APS was also distributed in two fractions, with molecular weights of 8.18 × 10^6^ Da and 2.21 × 10^4^ Da, respectively.

The HPLC chromatograms of the standard monosaccharides, TMP, and APS are presented in Figure 1D. The results revealed that TMP was predominantly composed of Man, Rha, GalA, Gal, Xyl, and Fuc, with a relative molar ratio of 1.25:1.75:2.83:8.33:4.83:1. In contrast, APS mainly consisted of Man, Rib, Rha, GlcA, GalA, Glc, Gal, Xyl, and Ara, with a relative molar ratio of 1:1.36:1.43:2.93:2.64:2.08:4.21:3.36:7.14.

As shown in Figure 1E, TMP and APS exhibited markedly distinct micromorphologies. TMP displayed an irregular flaky shape with a rough surface, whereas APS presented relatively smooth, elongated, or fibrous structures with interstructural gaps and slightly curled edges.

As shown in Figure 1F, neither polysaccharide exhibited any obvious sharp crystalline peaks within the diffraction angle range of 10°–50°. This indicated that purified TMP and APS existed predominantly in an amorphous state with low crystallinity, conforming to the structural characteristics typical of most naturally extracted polysaccharides.

### 3.2 TMP + APS ameliorates hepatic injury, fibrosis, and intestinal barrier damage

Before the commencement of modeling, no statistically significant differences in body weight were observed among the groups (Figure 2A). After the initiation of the drug administration period, the body weight gain in the Mod group slowed or even exhibited a declining trend, whereas the body weight changes in all treatment groups were improved compared with that in the Mod group. Notably, the TMP + APS group displayed a more pronounced improvement than the TMP and APS monotherapy groups. These results suggested that the combination of TMP and APS could, to a certain extent, alleviate the body weight decline in HF mice. The changes in liver index are presented in Figure 2B. Compared with the Con group, the liver index in the Mod group was significantly elevated (*P* < 0.01). Compared with the Mod group, the liver indices in the Pos1, Pos2, and TMP + APS groups were significantly decreased (*P* < 0.05). Importantly, the liver index of TMP + APS group was significantly lower than those of both theTMP group and APS group (P < 0.05), whereas the TMP and APS monotherapy groups only showed a mild decreasing tendency without statistical significance relative to Mod (P > 0.05). These results indicated that TMP + APS could improve the liver index in HF mice.

**FIGURE 2:**
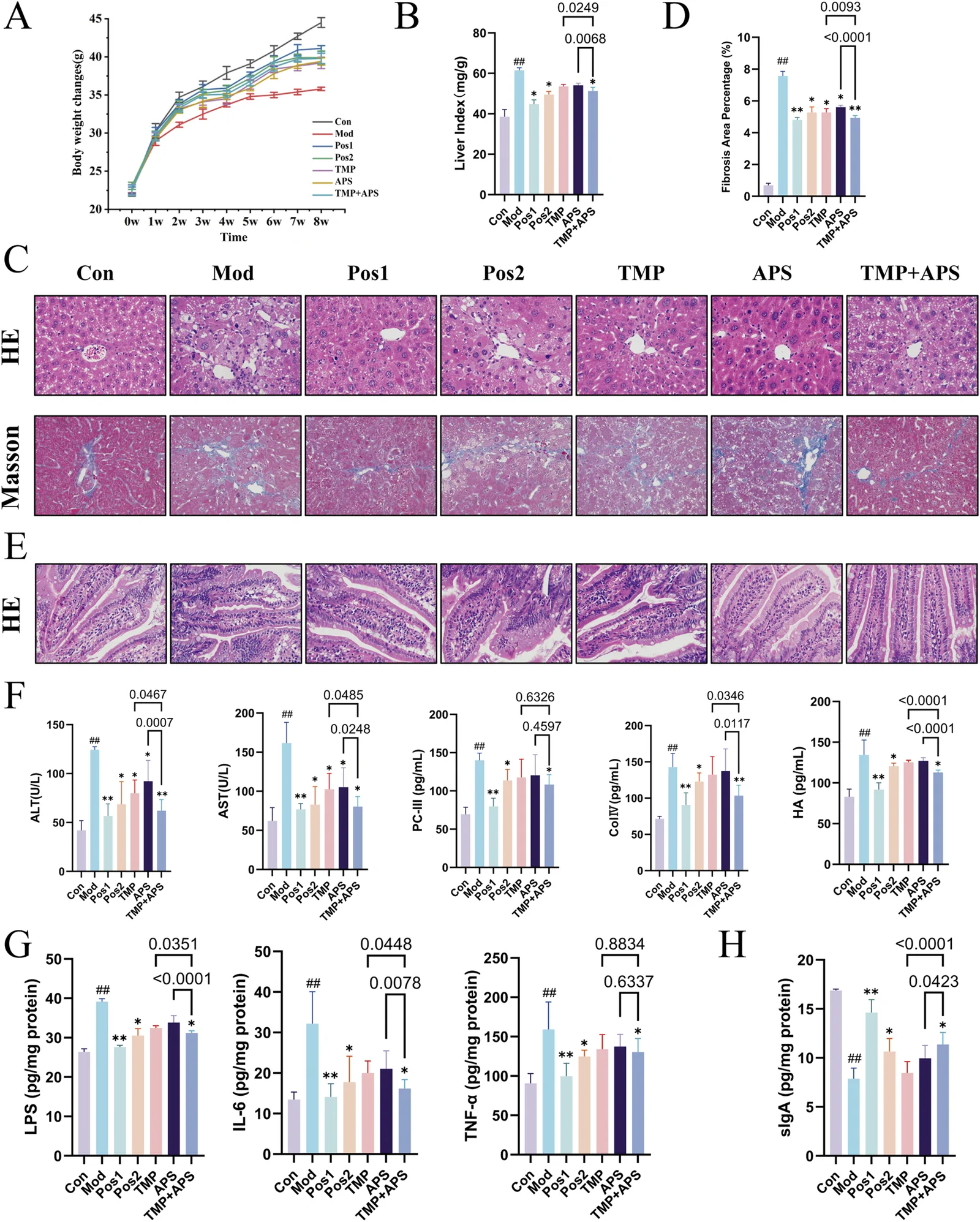
Combined treatment with TMP and APS ameliorates CCl_4_-induced hepatic injury, fibrosis, and intestinal barrier damage in mice. **(A)** Body weight changes of mice in each group during the experimental period. **(B)** Liver index of mice in each group (n = 10). **(C)** Histopathological changes in mouse liver tissues observed by H&E and Masson trichrome staining (Original magnification, ×400). **(D)** Quantitative analysis of hepatic fibrosis area percentage in each group. **(E)** Histopathological changes in mouse ileum tissues observed by H&E staining (Original magnification, ×200, n = 5). **(F)** Serum levels of ALT, AST, HA, PCIII, and Col IV in each group. **(G)** Hepatic levels of LPS, IL-6, and TNF-α in each group. **(H)** Ileal sIgA level in each group. Data are presented as the mean ± SD. #*p* < 0.05, ##*p* < 0.01 compared with the control (Con) group; *p < 0.05, **p < 0.01 compared with the model (Mod) group. The horizontal lines with exact p-values connecting the TMP, APS, and TMP + APS groups indicate direct pairwise comparisons among these treatment groups.

The results of H&E and Masson staining of liver tissues are shown in Figure 2C. Compared with the Con group, mice in the Mod group exhibited disordered liver architecture, with marked hepatocyte necrosis, inflammatory infiltration, and abnormal arrangement, confirming the successful establishment of the hepatic fibrosis model. After drug intervention, liver injury was alleviated to varying degrees in all treatment groups. Among them, the Pos1, Pos2, and TMP + APS groups indicated the most remarkable improvements in hepatocyte morphology and structural arrangement, approaching that of normal tissue. Although the TMP and APS groups also showed some degree of improvement, localized inflammatory infiltration and structural disorganization were still observed. These findings indicated that the combination of TMP and APS attenuated CCl_4_-induced hepatic tissue injury.

Masson staining of the liver tissues revealed that, compared with the Con group, the Mod group exhibited disrupted hepatic architecture with the appearance of pseudolobules and extensive hyperplastic collagen fibers. Following drug intervention, collagen fiber deposition was alleviated to varying degrees across all treatment groups. Among them, collagen deposition and fibrous hyperplasia were markedly reduced in the Pos1, Pos2, and TMP + APS groups, whereas only sporadic attenuation of collagen deposition and a modest decrease in fibrous hyperplasia were observed in the TMP and APS groups. These results indicated that TMP + APS ameliorated hepatic tissue injury in HF mice. Quantitative analysis of the collagen volume fraction (Figure 2D) further indicated that the TMP + APS group significantly decreased the hepatic fibrosis ratio compared with the model group (*P* < 0.05), and its efficacy was superior to that of either single-polysaccharide group (*P* < 0.05). Collectively, these results indicated that the combination of TMP and APS reduced fibrosis severity.

H&E staining of ileal tissues is presented in Figure 2E. Compared with the Con group, the ileal mucosal architecture in the Mod group was damaged, as evidenced by villous atrophy, a reduced number of epithelial cells, and loose cellular arrangement. After pharmacological treatment, ileal mucosal injury was ameliorated to varying degrees in all treatment groups. The restoration of mucosal structure was most pronounced in the Pos1, Pos2, and TMP + APS groups, characterized by improved villous morphology and a tighter arrangement of epithelial cells. In contrast, the TMP and APS groups exhibited limited improvement, with sparse cellular arrangement still observable in certain regions. These findings imply that combined TMP and APS treatment may be associated with greater improvement in hepatic fibrosis-related intestinal mucosal injury.

To evaluate the therapeutic effects of TMP and APS on carbon tetrachloride (CCl_4_)-induced hepatic fibrosis, key biomarkers in serum and tissues were measured. Compared with the Con group, mice in the Mod group exhibited significantly elevated serum levels of liver function indicators (ALT and AST) and hepatic fibrosis markers (HA, PCIII, and Col IV), confirming the successful establishment of hepatic injury and fibrosis. Concurrently, hepatic levels of the inflammatory cytokines LPS, IL-6, and TNF-α were markedly increased in the Mod group, whereas the ileal content of secretory immunoglobulin A (sIgA), was significantly suppressed, suggesting intensified systemic inflammation and impaired intestinal barrier function. All treatment groups (TMP, APS, and TMP + APS) significantly reduced serum ALT and AST levels compared with the Mod group (*P* < 0.05, Figure 2F). In terms of ameliorating hepatic fibrosis and liver inflammation, the combination of TMP and APS exhibited a more pronounced beneficial trend: the TMP + APS group significantly decreased serum levels of HA, PC III, and Col IV as well as hepatic levels of LPS, IL-6, and TNF-alpha (*P* < 0.05, Figure 2G), whereas the TMP and APS monotherapies did not achieve statistical significance for several of these indicators (*P* > 0.05). In terms of serum liver injury indicators ALT and AST, fibrosis markers Col IV and HA, hepatic LPS and IL-6, and ileal mucosal immune indicators sIgA, the therapeutic effect of TMP + APS was significantly better than that of using TMP or APS alone (all *P* < 0.05; Figure 2H). Overall, the combination treatment showed significantly stronger protective effects than either monotherapy across several, but not all, indicators of hepatic fibrosis, inflammation, and intestinal barrier function.

### 3.3 TMP + APS modulates gut microbiota composition and increase fecal short-chain fatty acid levels

To investigate the potential mechanisms underlying the combined anti-fibrotic effects of TMP and APS, 16S rRNA gene sequencing was performed on the colonic contents of mice. Analysis at the operational taxonomic unit (OTU) level (Figure 3A) revealed that the Mod group harbored the fewest unique OTUs, whereas the TMP + APS group shared a larger number of OTUs with the Con group, suggesting a correlation between combined polysaccharide treatment and partial restoration of baseline microbial community structure. Alpha diversity analysis (Figure 3B) demonstrated that, compared with the Con group, the Mod group exhibited significantly decreased Chao1, Observed species, and Shannon indices (*P* < 0.01) and an increased Simpson index, indicating that HF induced a profound reduction in the species richness and diversity of the gut microbiota. TMP + APS combination therapy significantly improved this trend, and the recovery of Chao1, Observed species and Shannon indices was significantly more pronounced than TMP monotherapy and APS monotherapy (P < 0.05), with an overall magnitude of improvement superior to single-polysaccharide intervention. Beta diversity analysis using weighted UniFrac distance was visualized via PCoA (Figure 3C). PERMANOVA with 999 permutations further confirmed significant overall dissimilarity in gut microbial community structure between the Con and Mod groups (*p* < 0.001). The microbial community of the TMP + APS group shifted toward the profile observed in the Con group, with samples clustering closer to control samples in PCoA ordination. At the phylum level (Figure 3D), the HF mice exhibited typical dysbiosis features: the relative abundance of Firmicutes was markedly increased, whereas that of Bacteroidetes was decreased, resulting in a sharp elevation of the Firmicutes/Bacteroidetes (F/B) ratio (*P* < 0.01). TMP + APS combination therapy effectively corrected this imbalance and significantly reduced the F/B ratio (*P* < 0.05, Figure 3E), the F/B ratio in TMP + APS group was significantly lower than that in TMP and APS groups (P < 0.01), shifts in this ratio can only be interpreted as one correlative microbial signature. To systematically characterize the gut microbial dysbiosis triggered by CCl_4_-induced hepatic fibrosis, pairwise LEfSe analysis was applied to compare the blank control and model groups (Figure 4A). In the Con group, multiple taxa were identified as characteristic biomarkers, including phylum Patescibacteria, class Saccharimonadia, order Saccharimonadales, family Saccharimonadaceae, genus Candidatus_Saccharimonas, as well as Blautia, *Bacteroides*, and *Streptococcus* within Firmicutes and Bacteroidetes. These taxa potentially associated with SCFA metabolism or intestinal homeostasis. In contrast, the Mod group was uniquely enriched in class Bacilli, family Staphylococcaceae, and genus *Staphylococcus*. Global multi-group LEfSe analysis covering all experimental groups was conducted to screen treatment-specific microbial biomarkers (Figure 4B). Consistent with pairwise comparison results, the Con group exhibited abundant differentially enriched microbial biomarkers dominated by Pelomonas, Ruminococcus_gnavus_group, Arsenicibacter, and phylum Cyanobacteria/Deferribacterota. Notably, the TMP + APS group was characterized by differential enrichment of several Actinobacteria-associated taxa, including Corynebacterium, Brachybacterium, and Aerococcus. The biological relevance of these taxa to intestinal inflammation and hepatic fibrosis remains to be clarified.

**FIGURE 3:**
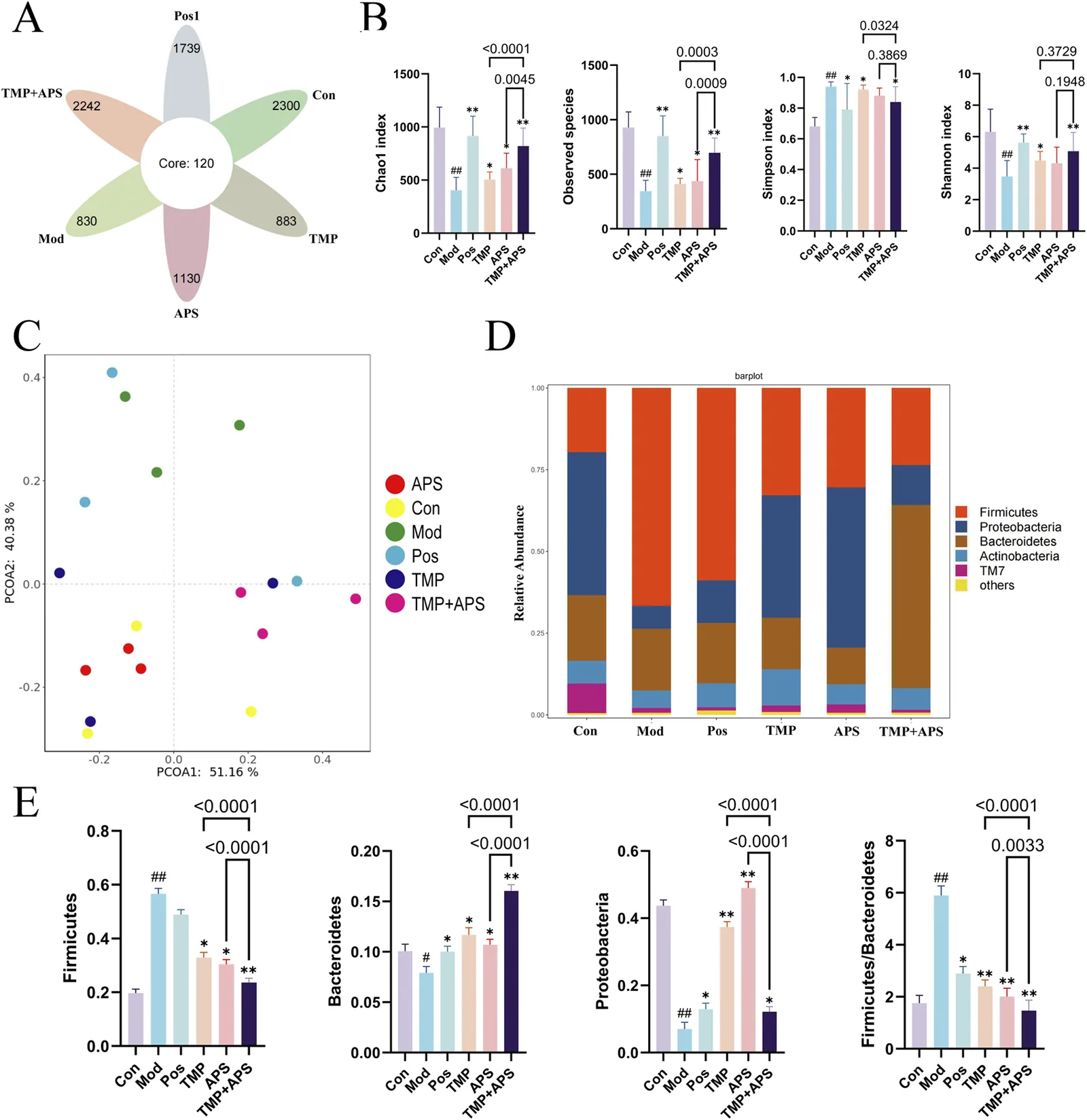
Combined TMP and APS treatment remodels gut microbiota diversity and community structure in CCl_4_-induced fibrotic mice (n = 10). **(A)** Venn diagram of operational taxonomic units (OTUs) of the gut microbiota in each group. **(B)** Bar charts of alpha diversity indices, including the Chao1, Observed species, Shannon, and Simpson indices, for the gut microbiota in each group. **(C)** Principal coordinate analysis (PCoA) based on weighted UniFrac distance. **(D)** Relative abundance of the gut microbiota at the phylum level. **(E)** Relative abundance analysis of the gut microbiota in each group. #*p* < 0.05, ##*p* < 0.01 vs. the Con group; *p < 0.05, **p < 0.01 vs. the Mod group. The horizontal lines with exact p-values connecting the TMP, APS, and TMP + APS groups indicate direct pairwise comparisons among these treatment groups.

**FIGURE 4:**
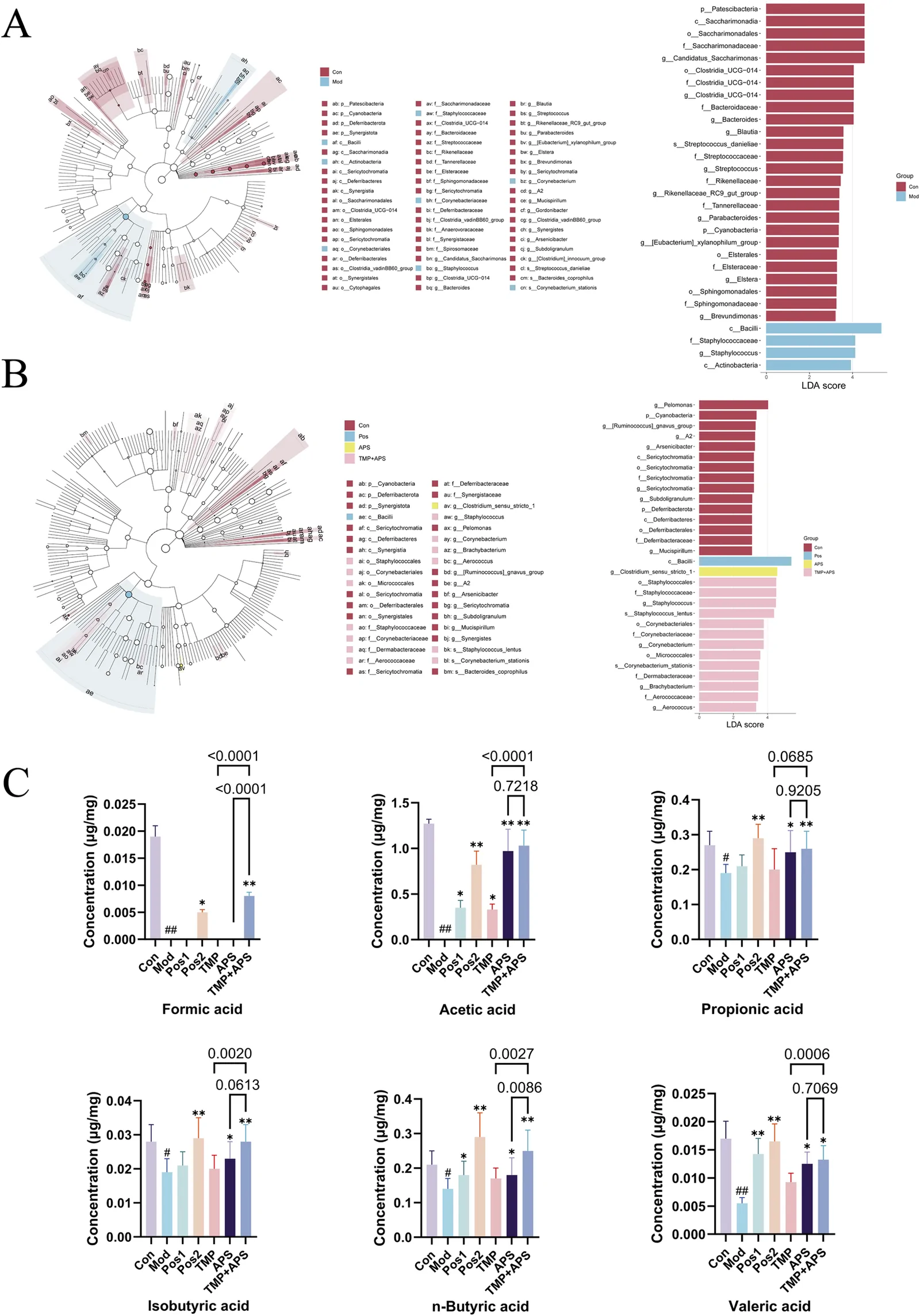
Gut microbiota alterations and fecal short-chain fatty acid profiles among experimental groups. **(A)** Pairwise LEfSe comparison between Con and Mod groups. The cladogram depicts the taxonomic distribution of differential microbial taxa between normal and fibrotic mice, and the LDA bar chart displays taxa with LDA score (top30). **(B)** Multi-group LEfSe analysis across all seven experimental groups. The cladogram depicts the taxonomic distribution of differential microbial taxa, and the LDA bar chart displays taxa with LDA score (top30) enriched in each group. **(C)** Combined administration of *Taraxacum mongolicum* Polysaccharides and Astragalus Polysaccharides elevates fecal short-chain fatty acid levels in mice. The concentrations of formic acid, acetic acid, propionic acid, isobutyric acid, n-butyric acid, and valeric acid in Fecal samples in each group were detected by high-performance liquid chromatography (HPLC). #*p* < 0.05, ##*p* < 0.01 vs. the Con group; *p < 0.05, **p < 0.01 vs. the Mod group, n = 6. The horizontal lines with exact p-values connecting the TMP, APS, and TMP + APS groups indicate direct pairwise comparisons among these treatment groups.

Short-chain fatty acids (SCFAs), key metabolic products of the gut microbiota, participate in maintaining intestinal barrier integrity and modulating systemic inflammation. We measured the SCFA contents in mouse fecal samples by HPLC (Figure 4C). The results demonstrated that, compared with the Con group, the levels of the major beneficial SCFAs—acetate, propionate, and butyrate—were significantly or highly significantly decreased in the Mod group (*P* < 0.05 or *P* < 0.01), indicating that HF was accompanied by reduced fecal SCFA levels, reflecting altered metabolic function of the gut microbiota. Following therapeutic intervention, the TMP + APS combination group significantly improved this trend, restoring the levels of acetate, propionate, and butyrate (all *P* < 0.05 vs. Mod). The concentration of isovaleric acid was below the detection limit in all groups, and there was no statistical difference between the groups. To avoid redundancy in the main graph, this data was not displayed. The concentrations of these key beneficial SCFAs in TMP + APS group were significantly higher than those in TMP monotherapy or APS monotherapy groups (P < 0.05).

### 3.4 TMP + APS reduces hepatic expression of TLR4/MyD88/NF-κB pathway components

Given that intestinal dysbiosis often facilitates endotoxin translocation and subsequently activates the hepatic innate immune system, we investigated the expression changes of the key hepatic inflammatory signaling pathway—TLR4/MyD88/NF-κB. To elucidate the molecular mechanisms underlying its anti-inflammatory and anti-fibrotic effects, we detected the protein expression of critical components of the TLR4/MyD88/NF-κB pathway in the liver by Western blot. As shown in Figures 5A,B, compared with the Con group, the protein expression levels of TLR4, MyD88, and NF-κB p65 in the liver of Mod group mice were all highly significantly upregulated (P < 0.01), indicating upregulation of the expression of these pathway components at protein and mRNA levels. All therapeutic interventions inhibited this aberrant activation to varying degrees. Compared with the Mod group, the protein expression of TLR4, MyD88, and NF-κB p65 was significantly downregulated in all treatment groups (Pos1, Pos2, TMP, APS, and TMP + APS) (*P* < 0.05). Notably, the combined TMP and APS group exhibited significantly lower protein and mRNA levels of TLR4, MyD88 and NF-κB p65 than the TMP monotherapy and APS monotherapy groups (P < 0.05).

**FIGURE 5:**
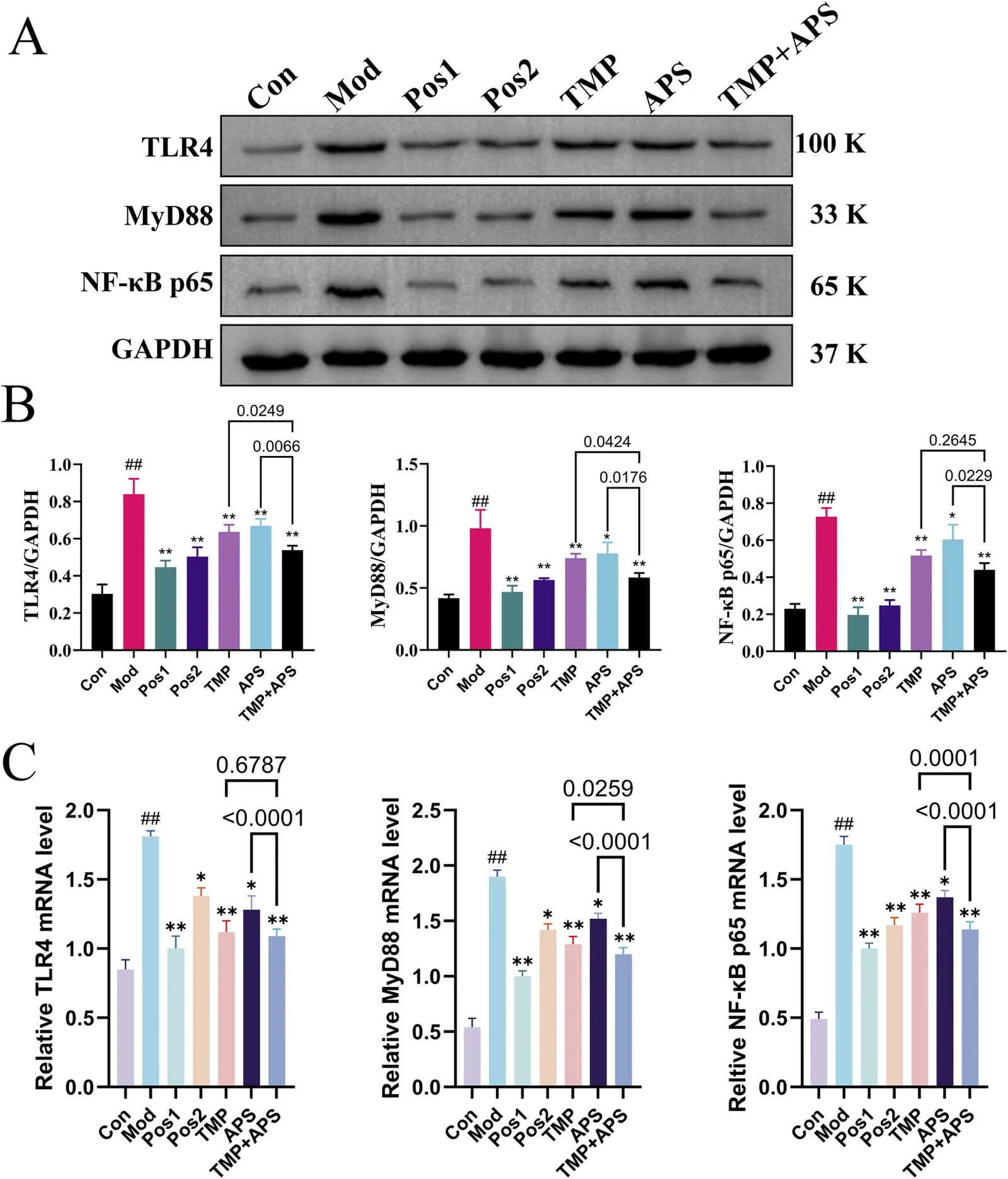
Combined treatment with *Taraxacum mongolicum* polysaccharide and Astragalus polysaccharide downregulates hepatic expression of TLR4/MyD88/NF-κB pathway-related genes and proteins in mice. **(A)** Representative Western blot images of TLR4, MyD88, and NF-κB p65 protein expression. **(B)** Bar charts of the relative protein expression levels of TLR4, MyD88, and NF-κB p65. **(C)** Relative mRNA expression levels of TLR4, MyD88, and NF-κB p65 determined by qRT-PCR. Data are expressed as mean ± SD. #*p* < 0.05, ##*p* < 0.01 vs. the Con group; *p < 0.05, **p < 0.01 vs. the Mod group. The horizontal lines with exact p-values connecting the TMP, APS, and TMP + APS groups indicate direct pairwise comparisons among these treatment groups.

The transcriptional levels of key genes in the TLR4/MyD88/NF-κB signaling pathway were analyzed by qRT-PCR. As shown in Figure 5C, compared with the Con group, the mRNA expression levels of TLR4, MyD88, and NF-κB p65 in the liver of Mod group mice were significantly upregulated (*P* < 0.01), indicating that this pathway was activated at the transcriptional level during hepatic fibrogenesis. After pharmacological treatment, compared with the Mod group, the Pos1 group exhibited highly significant inhibition of the expression of these three genes (*P* < 0.01), and the Pos2, TMP, APS, and TMP + APS groups all significantly reduced their mRNA levels (*P* < 0.05). These results were largely consistent with the Western blot findings, suggesting that the reduced activation of the TLR4/MyD88/NF-κB p65 signaling pathway is associated with the anti-fibrotic effects of TMP and APS, although causal validation through pathway inhibition experiments is still needed.

### 3.5 Correlation among gut microbiota, SCFAs, and hepatic inflammatory signaling

These correlation analyses reveal statistical associations among the gut microbiota, SCFA levels, and hepatic inflammatory signaling but do not establish causal relationships. Based on hepatic signaling pathway indicators, inflammatory factors, short-chain fatty acids, and gut microbiota, Spearman correlation analysis was performed to examine the correlations among these variables. The results demonstrated that the protein expression levels of hepatic TLR4, MyD88, and NF-κB p65 were significantly positively correlated with hepatic LPS content and the levels of the pro-inflammatory cytokines TNF-α and IL-6. Conversely, these three key pathway proteins were significantly negatively correlated with the fecal levels of the major SCFAs-acetate, propionate, and butyrate (Figure 6A). Correlation analysis between phylum-level gut microbiota abundance and host phenotypic indicators revealed that the relative abundance of Firmicutes was significantly positively correlated with the levels of LPS, TNF-α, and IL-6, and significantly negatively correlated with the levels of SCFAs such as formic acid, acetic acid, and propionic acid. Propionic acid was positively correlated with Bacteroidetes, TNF-α was negatively correlated with the TM7 phylum, and formic acid was positively correlated with the TM7 phylum (Figure 6B). It should be noted that these statistical relationships only reflect simultaneous variation of detected indicators and do not prove causal interactions.

**FIGURE 6:**
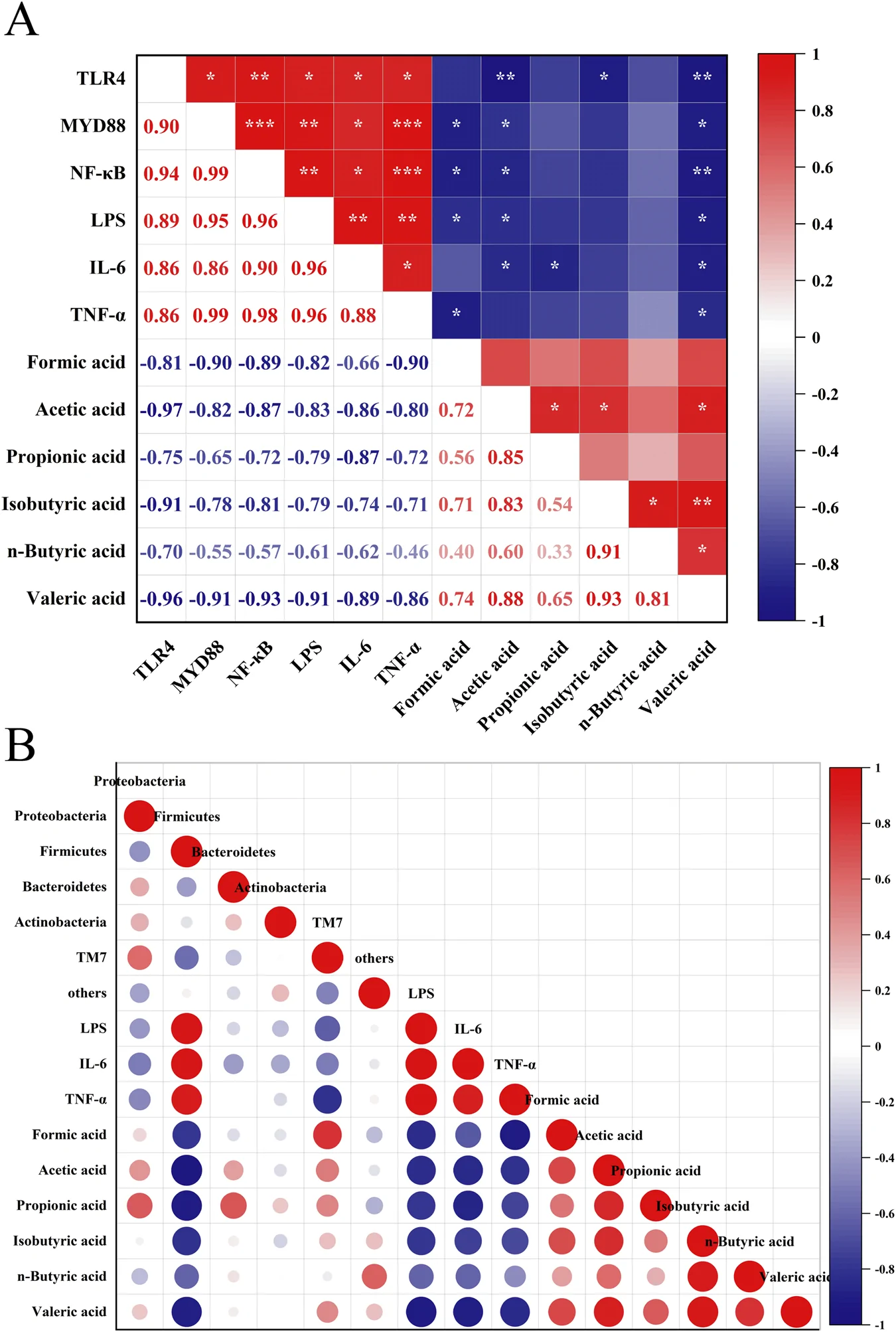
**(A)** Spearman correlation heatmap of hepatic TLR4/MyD88/NF-κB pathway proteins, inflammatory cytokines, LPS, and SCFAs. **(B)** Correlation plot among gut microbiota phyla, inflammatory markers, and short-chain fatty acids.

## 4 Discussion

Our results demonstrated that this herbal pair (TMP and APS) significantly ameliorated hepatic histopathological injury, reduced collagen deposition, and decreased serum levels of ALT, AST, and fibrosis markers (HA, PC III, and Col IV). Additionally, it reduced the hepatic expression of pro-inflammatory cytokines (TNF-α and IL-6) and LPS. The observed elevation in sIgA was associated with improved intestinal barrier phenotypes; however, the present data do not allow us to conclude that elevated sIgA directly enhances intestinal immune barrier function. To compare the therapeutic efficacy of the combined polysaccharide intervention with that of each monotherapy, we adopted a fixed-total-dose design. The combination group received a total dose of 200 mg/kg (100 mg/kg TMP +100 mg/kg APS), and each monotherapy group received 200 mg/kg of the respective single agent. This design ensures that any observed advantage of the combination cannot be attributed to a higher total dose. This fixed-total-dose design demonstrates only that the combined intervention produces superior therapeutic outcomes compared with each monotherapy at equal total doses; it does not establish definitive mechanistic synergy or complementarity between TMP and APS. Furthermore, we did not formally quantify synergistic effects using the Chou-Talalay combination index method, which represents a limitation of the present study. Nevertheless, pairwise statistical comparisons revealed that TMP + APS treatment exerted significantly stronger effects on fibrosis, intestinal barrier protection, microbiota regulation, and inflammation than either TMP or APS monotherapy. These superior effects were reflected by multiple core indicators, including liver index; serum biochemical parameters (ALT and AST); serum fibrosis markers (Col IV and HA); hepatic LPS levels; the pro-inflammatory cytokine IL-6; ileal sIgA; gut microbiota alpha diversity; the Firmicutes/Bacteroidetes (F/B) ratio; fecal short-chain fatty acid (SCFA) levels; and hepatic expression of TLR4/MyD88/NF-κB pathway components.

Gut-liver axis disruption is increasingly recognized as a key driver of hepatic fibrosis development and progression (Qin et al., 2024). We observed that HF mice displayed severe intestinal dysbiosis, characterized by significantly reduced alpha diversity (as reflected by Chao1 and Shannon indices), an abnormally elevated Firmicutes/Bacteroidetes (F/B) ratio, and a marked decrease in the beneficial SCFAs, including acetate, propionate, and butyrate. These observations are consistent with previous reports of gut microbiota dysbiosis in the context of liver diseases (Chang et al., 2023; Sun et al., 2024; Cui et al., 2025). At the phylum level, Firmicutes and Bacteroidetes were the most abundant bacterial populations in the gut ecosystem. The model group exhibited an elevated F/B ratio, accompanied by decreased fecal SCFA concentrations. The relative abundances of Proteobacteria, Actinobacteria, and TM7 also differed markedly between the control and model groups. Compared with single-polysaccharide treatment, combined TMP and APS intervention shifted microbial diversity, the F/B ratio, and SCFA contents toward the levels observed in normal mice. Notably, APS treatment induced a relatively greater elevation in SCFA concentrations and ileal sIgA levels in this model, consistent with prior reports describing APS regulation of gut microecology and SCFA generation (Wei et al., 2023; Rong et al., 2025). In contrast, TMP treatment produced relatively more pronounced improvement in hepatic inflammatory markers in our experimental system (Zhang et al., 2016; Li Z. et al., 2024). Their biological actions likely overlap; however, any partial complementary effects should be regarded as a preliminary hypothesis requiring further verification. Consistent with these observations, co-administration of TMP and APS resulted in more favorable changes across a broader range of gut–liver axis indicators compared with either monotherapy. Firmicutes abundance was significantly positively correlated with hepatic levels of LPS, TNF-α, and IL-6, whereas Bacteroidetes abundance showed a positive correlation with propionate levels. The TMP + APS combination effectively reduced the F/B ratio and partially restored the microbial community structure. Given the positive correlation between Firmicutes abundance and hepatic LPS levels, the reduction in Firmicutes may contribute to the observed decrease in hepatic LPS. However, intestinal permeability, tight-junction protein expression, and portal or systemic endotoxin levels were not measured in this study. Therefore, the source (gut-derived vs. systemic) and translocation route of LPS cannot be definitively determined from the current data. In the LEfSe analysis, the genus *Staphylococcus* (phylum Firmicutes, class Bacilli) exhibited higher relative abundance in fibrotic model mice, whereas genera reported to correlate with SCFA biosynthesis, including Blautia and *Bacteroides*, were less abundant in the model group. Nevertheless, this differential taxon distribution alone cannot fully explain the reduced SCFA concentration and compromised intestinal barrier observed in model animals. Additionally, several Actinobacteria genera, including Corynebacterium and Brachybacterium, were identified as differentially abundant taxa characteristic of the TMP + APS group; however, their physiological functions related to hepatic fibrosis were not confirmed in the present study.

At the molecular level, we focused on the TLR4/MyD88/NF-κB axis, which serves as the core pathway connecting gut-derived endotoxins to hepatic inflammation. Western blotting and qRT-PCR analyses revealed elevated expression of TLR4, MyD88, and NF-κB p65 at both the protein and mRNA levels in HF model mice. Conversely, combined TMP and APS administration downregulated the expression of these pathway components, with the combination group showing a more pronounced inhibitory effect than either monotherapy group. TLR4 functions as the primary pattern recognition receptor for LPS, and its ligation initiates MyD88-dependent NF-κB signaling, which subsequently promotes the transcription of pro-inflammatory cytokines, including TNF-α and IL-6. This cascade is considered a critical trigger for hepatic stellate cell (HSC) activation and fibrogenesis (Gan et al., 2018). Notably, hepatic protein levels of TLR4, MyD88, and NF-κB p65 were negatively correlated with fecal concentrations of acetate, propionate, and butyrate, suggesting that SCFAs may suppress TLR4/NF-κB pathway activation. This inverse relationship is consistent with previous findings demonstrating that garlic polysaccharide and Angelica sinensis polysaccharide ameliorate alcoholic liver disease by modulating the gut microbiota and SCFA metabolism (Wang et al., 2018; He et al., 2025). Collectively, these observations further support the feasibility of polysaccharide-based interventions targeting the gut–liver axis for the treatment of hepatic fibrosis.

Several limitations of this study should be acknowledged. First, although Masson trichrome staining and serum fibrosis markers provide robust evidence for anti-fibrotic effects, direct assessment of hepatic stellate cell activation markers, such as α-SMA, and hepatic hydroxyproline content would further strengthen our conclusions. Second, the observed changes in the TLR4/MyD88/NF-κB pathway are associative rather than causal; pharmacological inhibition using TLR4-specific inhibitors (e.g., TAK-242) or genetic knockout models would be required to definitively establish that the TMP + APS combination acts through this pathway. Third, fecal SCFA measurements reflect colonic microbial metabolism but do not capture portal SCFA concentrations or hepatic tissue exposure. Fourth, 16S rRNA sequencing provides only genus-level taxonomic resolution; metagenomic and metatranscriptomic sequencing are needed to dissect the functional metabolic pathways of key butyrate-producing strains regulated by this polysaccharide combination. Fifth, endotoxin contamination in the purified TMP and APS polysaccharide samples was not assessed, and therefore its potential interference with the observed inflammatory phenotypes cannot be completely excluded. Finally, although correlation analyses revealed close associations among gut microbiota composition, SCFA levels, and hepatic TLR4/MyD88/NF-κB signaling, these findings remain at a correlative level and await causal validation. Future studies integrating fecal microbiota transplantation, pathway-specific blockade models, and multi-omics metabolomics will be required to establish a causal link between the TMP + APS-reshaped gut microbiota and the alleviation of hepatic fibrosis.

## 5 Conclusion

Our data indicate that combined administration of TMP and APS exerts greater therapeutic outcomes against CCl_4_-triggered hepatic fibrosis compared with TMP or APS monotherapy. Multiple detected indices showed correlations between combined administration and altered gut microbial profiles, elevated fecal SCFA concentrations, as well as lowered hepatic TLR4, MyD88 and NF-κB p65 transcript and protein abundance. The causal regulatory links between these intestinal and hepatic phenotypic shifts remain unvalidated and require further targeted research.

## Data Availability

The 16S rRNA gene sequencing data presented in this study have been deposited in the NCBI Sequence Read Archive (SRA) under BioProject accession number PRJNA1520270.
