# Supplementary material for: Combined Taraxacum mongolicum and Astragalus polysaccharides attenuate CCl4-induced hepatic fibrosis through modulation of the TLR4/MyD88/NF-κB signaling pathway and gut microbiota composition
Source: Front Pharmacol. 2026 Sep 17;17:1904671. doi: 10.3389/fphar.2026.1904671 (PMC13627329; doi:10.3389/fphar.2026.1904671)
Supplement: Supplementary file 1 [file Table1.docx]

Supplementary Material

**Supplementary Figure 1** High performance liquid phase diagram of short chain fatty acids in feces of mice in each group. Notes: a. Mixed standards (1. Formic acid; 2. Acetic acid; 3. Propionic acid; 4. Isobutyric acid; 5. N-butyric acid; 6. N-valeric acid; 7. Isovaleric acid); b. Con group; c. Mod group; d. Pos1 group; e. Pos2 group; f. TMP group; g. APS group; h. TMP+APS co-administration group.

**Supplementary Method S1:** The calculation formula for polysaccharide extraction yield is as follows:

$$Y=\frac{m}{M}\times100\%$$

Where:

Y represents the extraction yield of polysaccharides (%);

m is the mass of dried polysaccharide (g);

M stands for the initial mass of raw medicinal material (g).

The extraction yield of TMP was 8.32 ± 0.07%, and the extraction yield of APS was 4.96 ± 0.12%.

**Supplementary Method S2:** Detailed Procedures for 16S rRNA Gene Sequencing and Bioinformatics Analysis

Total sequencing sample quantity: A total of 70 fecal samples (7 groups, 10 mice in each group) were sequenced using the Illumina NovaSeq 6000 platform for dual-end sequencing of the V3-V4 regions;

Sequencing read length and sample reads: The average original reads per sample was 58,600, with the minimum original sequencing quantity being 45,300;

Quality control and chimeric removal: Quality control was performed using QIIME2, with a Q20 filtering threshold, and the DADA2 algorithm was used to remove chimeras and generate ASVs. Traditional OTU clustering thresholds were not used;

Species annotation and normalization: The species annotation database was SILVA 138.1 version; All samples were diluted to 42,000 effective reads for analysis, and the Good's coverage of each sample was greater than 0.99, indicating sufficient sequencing depth;

Statistical verification of community differences between groups: In addition to the PCoA visualization, the PERMANOVA (Adonis test, with 999 permutations) statistical results were supplemented to confirm that the overall structure of the microbiota in the blank group and the model group was significantly separated (p < 0.001). The bacterial community structure of the combined group recovered significantly towards the normal group compared to the model group.

**Supplementary Method S3:** HPLC Conditions for Short-Chain Fatty Acid (SCFA) Analysis in Mouse Feces

Instrument: Agilent 1260 Infinity high-performance liquid chromatograph, equipped with a differential refractive index detector (RID-10A), with the detector temperature set at 37 ℃.

Column: Carbomix H-NP5:8% (7.8 mm × 300 mm, 5 μm), with a matching protective column (7.8 mm × 50 mm, 5 μm); the column temperature box temperature is 43 ℃.

Mobile phase: 2.5 mmol/L sulfuric acid aqueous solution; isocratic elution.

Flow rate: 0.7 mL/min; injection volume: 100 μL.

Internal standard: Before extraction of all fecal samples, 2-ethylbutyric acid (0.5 g/100 mL) was added as an internal standard to correct for injection errors and losses during the extraction process. The extraction recovery rates of each SCFA ranged from 85.2% to 94.7%.

Quantification and performance: A standard curve was plotted using the peak area ratio of SCFA to the internal standard. The 7 SCFAs showed good linearity within their corresponding concentration ranges (R^2^ ≥ 0.9990). The detection limit (LOD) and quantification limit (LOQ) ranges were 0.01 to 0.05 g/100 mL and 0.05 to 0.15 g/100 mL, respectively.

Sample normalization: The content of fecal SCFA was expressed in micrograms per milligram of wet feces, and the exact wet weight of each fecal sample was weighed before extraction. Samples from the same batch were analyzed in the same analytical sequence to minimize inter-batch variations.
